# Fructose-induced stress signaling in the liver involves methylglyoxal

**DOI:** 10.1186/1743-7075-10-32

**Published:** 2013-04-08

**Authors:** Yuren Wei, Dong Wang, Gretchen Moran, Andrea Estrada, Michael J Pagliassotti

**Affiliations:** 1 Department of Food Science and Human Nutrition, Colorado State University, 234 Gifford, Fort Collins, CO 80523-1571, USA

**Keywords:** Sucrose, Insulin resistance, Mitogen-activated protein kinase

## Abstract

**Background:**

Fructose produces hepatic insulin resistance in humans and animals. We have proposed that the selective metabolism of fructose by the liver can, under conditions of elevated fructose delivery, inflict a metabolic insult that is localized to the hepatocyte. The present study was designed to identify potential cellular effectors of this insult.

**Methods:**

Primary hepatocytes were incubated with 8 mM glucose and 0.12% inulin (G, n = 6) or 8 mM glucose, 0.12% inulin and 28 mU of inulinase (GF, n = 6) in the presence or absence of insulin for 0, 2, or 4 h.

**Results:**

GF produced fructose concentrations of ~0.7 mM over the 4 h experiment. GF induced phosphorylation of MKK7 and JNK, phosphorylation of serine307 on IRS-1, and reduced tyrosine phosphorylation of IRS-1 and -2. GF increased ceramide levels and reactive oxygen species (ROS); however inhibitors of ceramide synthesis or ROS accumulation did not prevent GF-mediated changes in MKK7, JNK or IRS proteins. GF increased cellular methylglyoxal concentrations and a selective increase in methylglyoxal recapitulated the GF-induced changes in MKK7, JNK and IRS proteins.

**Conclusions:**

We hypothesize that GF-mediated changes in stress signaling involve methylglyoxal in primary hepatocytes.

## Background

Fructose is an intriguing nutrient due, in part, to its selective hepatic metabolism. The annual per capita consumption of extrinsic or added sucrose and fructose has increased in the US population [1,2]. In rats, diets enriched in sucrose or fructose can produce hepatic insulin resistance independently of changes in body composition [3-5]. Sucrose-induced hepatic insulin resistance occurred concomitantly with elevated hepatic c-jun NH_2_-terminal kinase (JNK) activity, and normalization of JNK activity in isolated hepatocytes improved insulin-stimulated tyrosine phosphorylation of insulin receptor substrate (IRS) proteins and insulin suppression of glucose release [6]. The ingestion of a single, sucrose-enriched meal or elevation of portal vein fructose concentrations via fructose infusion in rats in vivo also increased hepatic JNK activity and phosphorylation of insulin receptor substrate-1 (IRS-1) on serine^307^, a downstream target of JNK [6]. Fructose infusions in humans can also induce hepatic insulin resistance [7]. The mechanisms leading to these changes remain unclear.

We have proposed that the selective metabolism of fructose by the liver can, under conditions of elevated fructose delivery, inflict a metabolic insult that leads to insulin resistance and involves the hepatocyte [8]. The aim of the present manuscript was to identify potential cellular effectors that mediate fructose-induced activation of stress signaling (JNK) and insulin resistance. Candidate mediators included ceramide, reactive oxygen species and methylglyoxal all of which can activate JNK and accumulate in the context of excessive carbohydrate metabolism [8-11].

## Materials and methods

### Materials

Glucose, inulin, inulinase, insulin, and methylglyoxal were purchased from Sigma Chemical Co (St. Louis, MO). Primary antibodies were purchased from Cell Signaling (Beverly, MA).

### Animals

Male Wistar Crl:(WI)BR rats (Charles River Laboratories, Wilmington, MA) weighing 120-150 grams were provided free access to a purified high-starch diet (Research Diets, Inc, New Brunswick, NJ) for 1 week. All procedures were reviewed and approved by the Colorado State University institutional animal care committee.

### Hepatocyte isolation and culture

Hepatocytes were isolated from rats by collagenase perfusion [12]. Viability, based on trypan blue exclusion, was > 90%. Cells were first incubated with Roswell Park Memorial Institute media (RPMI) 1640 containing 11 mmol/L glucose, 10^-7^ mol/L dexamethasone, 10^-7^ mol/L insulin on collagen-coated plates containing 5% FBS for 4 h. Following attachment, the media was changed to one containing RPMI, 8 mmol/L glucose, 10^-7^ mol/L dexamethasone, and 10^-8^ mol/L insulin. The following morning media was replaced by RPMI containing glucose but not dexamethasone or insulin. After a 4 h period, experimental treatments were performed in the absence or presence of insulin. Each experiment was performed in triplicate.

### Experimental model

To perform these studies, a fructose regenerating system developed by Phillips et al. [13] was employed. In brief, inulinase and inulin were used to generate fructose at a rate designed to match fructose utilization. This delivery system minimizes disturbances in ATP and redox status that result from exposing cells to high concentrations of fructose or to nutrient limitation which can easily occur with fructose [6,14,15]. Primary rat hepatocytes were incubated with 8 mM glucose and 0.12% inulin (G, n = 6) or 8 mM glucose, 0.12% inulin and ~28 mU inulinase (GF, n = 6) in the absence or presence of 1 nM insulin for 0, 2, or 4 hrs. In studies utilizing methylglyoxal in the media (MG, n = 6) incubations were performed with 8 mM glucose, 0.12% inulin, and methylglyoxal in the absence or presence of 1 nM insulin for 4 hrs.

#### Media analyses

Glucose and fructose concentrations were determined using standard enzymatic procedures [16]. Methylglyoxal was measured by an o-phenylenediamine (PD) method [17]. Perchloric acid-precipitated samples were supplemented with 100 mM o-PD and the quinoxaline derivative of methylglyoxal (2-methylquinozaline) and the quinoxaline internal standard (5-methylquinoxaline) were then measured by high-performance liquid chromatography (Millipore).

#### Immunoprecipitation and western blot analysis

Cells were processed using previously described procedures [6]. Immunoprecipitation of IRS-1 and IRS-2 was performed using Dynabeads Protein G (Novex, Life Technologies). Briefly, equivalent amounts of protein were incubated with antibodies against IRS-1 or IRS-2 followed by incubation with Dynabeads Protein G. Western blot analysis for IRS-1, IRS-2 and tyrosine phosphorylation of IRS-1 and IRS-2 proceeded as described below. Equal amounts of immunoprecipitate or protein were separated by SDS-PAGE, electrotransferred to Hybond-P membranes and membranes incubated with the antibodies described in the Results section. Detection was performed using enhanced chemiluminescence reagents (Santa Cruz Biotechnology, Santa Cruz, CA) and band intensity was analyzed by optical density (UVP Bioimaging system, Upland, CA).

### Analysis of Akt

Total and phosphorylated (serine 473) Akt was determined using the STAR (Signal Transduction Assay Reaction) ELISA kit (Millipore) per the manufacturer’s instructions. This kit is based on a solid phase sandwich enzyme-linked immunosorbent assay in which 96-well plates are coated with a monoclonal Akt antibody and following incubation with cell lysates Akt is detected using a specific rabbit anti-Akt1 antibody or phosphorylated Akt is detected using a specific rabbit anti-phospho-Akt (Ser473) antibody.

### Oxidative stress

2,7-dichlorofluorescein di-acetate (DCFH-DA) fluorescence was used to estimate oxidative stress [18,19]. Following treatment, cells were loaded with 5 μM DCFH-DA (Molecular Probes) using serum free media for 45 min at 37°C. Fluorescence was monitored with excitation and emission wavelengths of 490 and 535 nm, respectively. Data are reported as the fold increase in median fluorescence over control cells.

### Ceramide

Ceramide was determined by a modification of the diacylglycerol kinase assay using [γ-^32^P] ATP and quantitation of the radioactive spot corresponding to ceramide- 1-phosphate [20,21].

### Statistical analysis

All data are reported as the mean ± standard deviation. Two-way repeated measures ANOVA was used for data analysis with post-hoc analyses that included linear contrasts and Student-Newman-Keul’s test. An α-level of p < 0.05 was used for statistical significance.

## Results

### Role of ceramide and oxidative stress in fructose-mediated stress signaling

**Figure 1:**
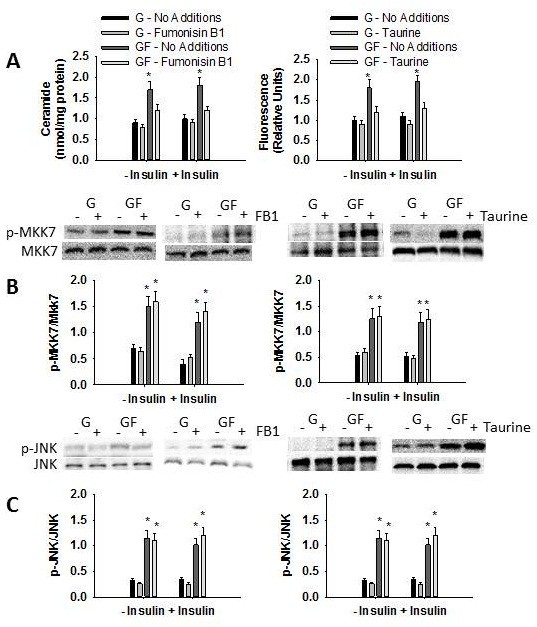
**Role of ceramide and oxidative stress in fructose-mediated stress signaling.** Ceramide (left) or DCF fluorescence (right) (**A**), phosphorylation of MKK7 (**B**), and phosphorylation JNK (**C**) in primary hepatocytes in response to glucose (G) or glucose and fructose (GF) in the absence (- insulin) or presence (+ insulin) of insulin. When present fumonisin B1 was at 50 uM and taurine at 1% w/v. Data in graphs are the mean ± SDEV for 6 independent experiments performed in triplicate. Experiments were 4 h in duration. * significantly different from G (p < 0.05).

**Figure 2:**
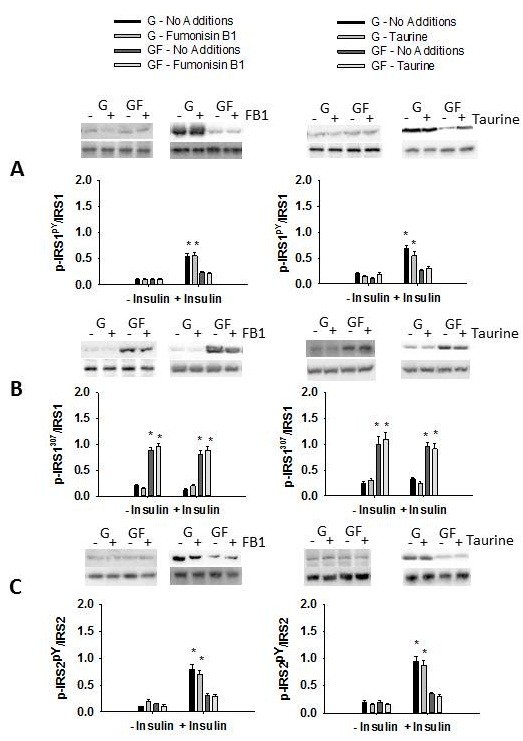
**Role of ceramide and oxidative stress in fructose-mediated insulin signaling.** Tyrosine phosphorylation of IRS1 (**A**), phosphorylation of serine 307 on IRS1 (**B**), and tyrosine phosphorylation IRS2 (**C**) in primary hepatocytes in response to glucose (G) or glucose and fructose (GF) in the absence (- insulin) or presence (+ insulin) of insulin. When present fumonisin B1 (left) was at 50 uM and taurine (right) at 1% w/v. Data in graphs are the mean ± SDEV for 6 independent experiments performed in triplicate. Experiments were 4 h in duration. * significantly different from G (p < 0.05).

**Figure 3:**
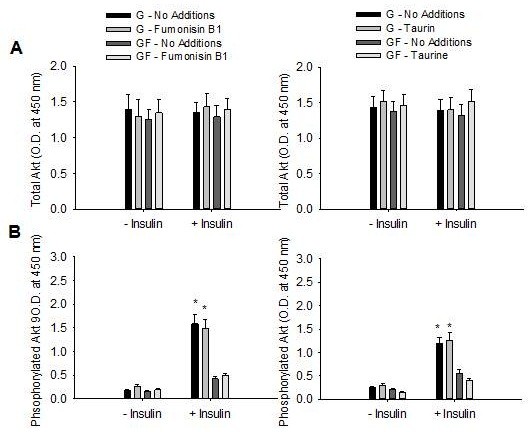
**Role of ceramide and oxidative stress in fructose-mediated phosphorylation of Akt.** Total (**A**) and phosphorylated (serine 473) (**B**) Akt in primary hepatocytes in response to glucose (G) or glucose and fructose (GF) in the absence (- insulin) or presence (+ insulin) of insulin. When present fumonisin B1 (left) was at 50 uM and taurine (right) at 1% w/v. Data in graphs are the mean ± SDEV for 6 independent experiments performed in triplicate. Experiments were 4 h in duration. * significantly different from G (p < 0.05).

The fructose regenerating system resulted in stable fructose concentrations of 0.66 ± 0.08 mM over the course of the 4 h experiment. Ceramide and reactive oxygen species can activate JNK [22-25]. Fructose delivery (GF) increased ceramide concentration and oxidative stress (Figure 1). Prevention of the fructose-mediated increase in ceramide or oxidative stress, using fumonisin B1 or taurine, respectively, did not mitigate the phosphorylation of MKK-7, JNK or serine^307^ of IRS-1 (Figures 1 and 2). In addition, the presence of fumonisin B1 or taurine did not increase insulin-stimulated tyrosine phosphorylation of IRS-1 or IRS-2 (Figure 2) or phosphorylation of Akt (Figure 3).

### Fructose-mediated changes in methylglyoxal

**Figure 4:**
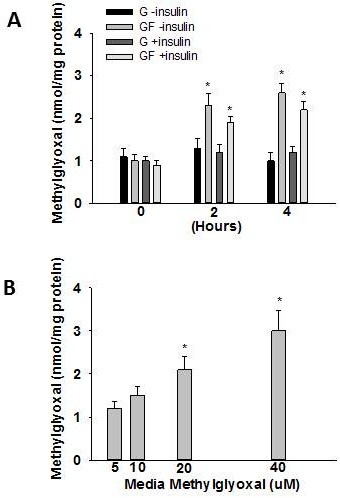
**Methylglyoxal concentration in response to fructose delivery and media methylglyoxal**. Liver cell methylglyoxal concentration in response to glucose (G) or glucose + fructose (GF) delivery in the absence or presence of insulin (**A**) or in response to varying media methylglyoxal concentrations (**B**). Data in graphs are the mean ± SDEV for 6 independent experiments performed in triplicate. Experiments were 4 h in duration. * significantly different from G (p < 0.05).

Fructose delivery (GF) increased methylglyoxal concentrations by approximately 100% in the absence or presence of insulin at both 2 and 4 h (Figure 4). In separate experiments primary hepatocytes were incubated with varying concentrations of methylglyoxal in the media in order to determine what media concentration elicited a similar cellular methylglyoxal concentration to that observed with fructose (i.e. ~2 nmol/mgprotein). A media methylglyoxal concentration of 20 uM resulted in liver cell methylglyoxal concentrations of ~2 nmol/mg protein (Figure 4).

### Methylglyoxal recapitulates the effects of fructose on stress signaling

**Figure 5:**
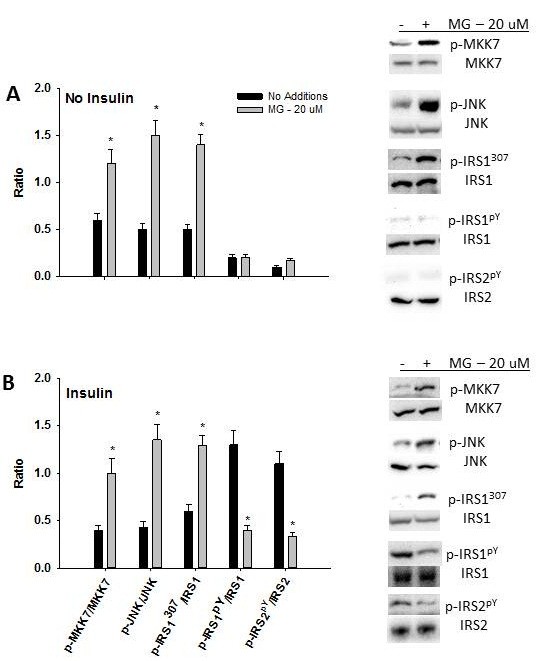
**Stress and insulin signaling in primary hepatocytes**. Phosphorylation of MKK7, JNK, serine 307 of IRS-1 and tyrosine phosphorylation of IRS-1 and IRS-2 in primary hepatocytes in response to methylglyoxal in the absence (**A**) or presence (**B**) of insulin. Data in graphs are the mean ± SDEV for 6 independent experiments performed in triplicate. * significantly different from No Additions (p < 0.05).

Incubation of primary hepatocytes with methylglyoxal (media concentration of 20 uM) for 4 h increased the phosphorylation of MKK7, JNK and serine^307^ of IRS-1 in the absence and presence of insulin (Figure 5). Methylglyoxal also reduced insulin-stimulated tyrosine phosphorylation of IRS-1 and IRS-2 (Figure 5).

### N-acetyl cysteine (NAC) reduces methylglyoxal and fructose-mediated stress signaling

**Figure 6:**
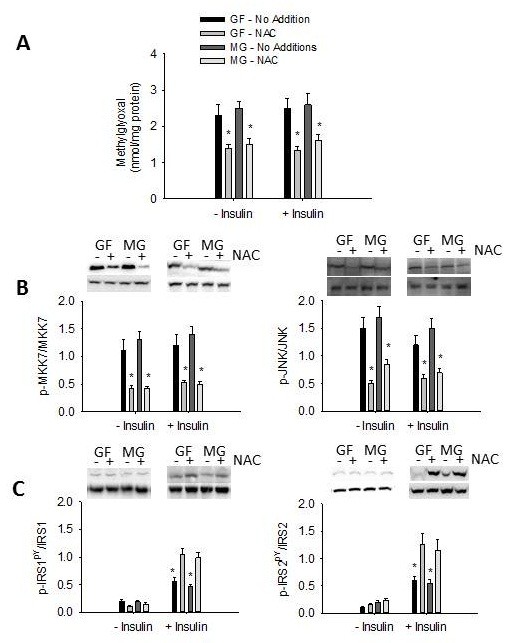
**Effects of N-acetyl cysteine on fructose- and methylglyoxal- mediated stress and insulin signaling in primary hepatocytes**. Methylglyoxal concentration (**A**), phosphorylation of MKK7 and JNK (**B**), and tyrosine phosphorylation of IRS-1 and IRS-2 (**C**) in primary hepatocytes incubated in the presence of fructose delivery (GF) or methylglyoxal in the media (MG) in the absence (No Additions) or presence of NAC (20 uM). Data in graphs are the mean ± SDEV for 4 independent experiments performed in triplicate. * significantly different from No Additions (p < 0.05).

**Figure 7:**
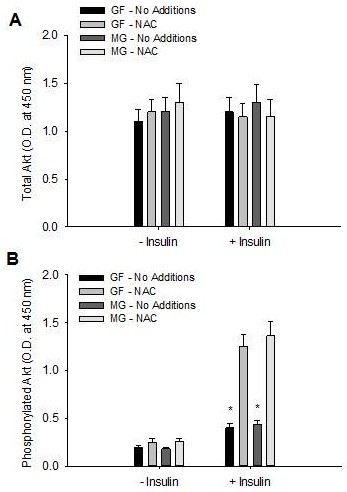
**Effects of N-acetyl cysteine on fructose- and methylglyoxal- mediated phosphorylation of Akt**. Total (**A**) and phosphorylated (serine473) (**B**) Akt in primary hepatocytes incubated in the presence of fructose delivery (GF) or methylglyoxal in the media (MG) in the absence (No Additions) or presence of NAC (20 uM). Data in graphs are the mean ± SDEV for 4 independent experiments performed in triplicate. * significantly different from No Additions (p < 0.05).

NAC is an antioxidant and methylglyoxal scavenger [26,27]. Incubation of primary hepatocytes with NAC over a 4 h period reduced fructose- and methylglyoxal-mediated increases in methylglyoxal, phosphorylation of MKK7, JNK and serine^307^ of IRS-1 in the absence and presence of insulin (Figure 6). The presence of NAC also increased tyrosine phosphorylation of IRS-1 and IRS-2 (Figure 6), and phosphorylation of Akt (Figure 7).

## Discussion

The present study, similar to our previous studies [6], demonstrates that increased fructose delivery activates MKK7 and JNK, and modifies the phosphorylation state of IRS-1 in rat primary hepatocytes. New data demonstrate that fructose delivery increased liver cell methylglyoxal concentrations within a time frame consistent with modifications in the phosphorylation state of MKK7, JNK and IRS-1. In addition, selective elevation of cellular methylglyoxal concentrations recapitulated the effects of fructose on these proteins. Finally, when the increase in methylglyoxal was prevented using N-acetyl cysteine, fructose-mediated changes in stress and insulin signaling were reduced. These data are consistent with the notion that acute effects of fructose delivery on stress signaling may be mediated by changes in cellular methylglyoxal.

Fructose delivery to and metabolism in the hepatocyte generates a signal that culminates in the activation of JNK, phosphorylation of serine^307^ of IRS-1 and reduced insulin-stimulated tyrosine phosphorylation of IRS-1 and IRS-2 [6]. We have hypothesized that this response to fructose delivery results from the burden of fructose metabolism [28]. However, the intrahepatic signal(s) that mediate fructose-induced stress signaling and modulation of insulin signaling have not been identified. In the present study, we examined three potential intrahepatic signals that can promote JNK activation and modulation of IRS-1 phosphorylation; ceramide, oxidative stress and methylglyoxal [11,23,25,29]. Although fructose delivery increased all three of these signals, inhibition of the increase in ceramide using fumonisin B1, or oxidative stress, using taurine, did not reduce fructose-mediated effects on stress or insulin signaling. In contrast, experiments in which cellular methylglyoxal concentrations were selectively elevated to levels observed with fructose delivery resulted in the activation of stress signaling and reductions in insulin signaling. In addition, the presence of N-acetyl cysteine, which effectively reduced cellular methylglyoxal concentrations, reduced fructose- and methylglyoxal-mediated effects on stress and insulin signaling. These data are consistent with the notion that acute effects of fructose delivery on hepatocyte stress and insulin signaling are mediated by methylglyoxal.

Increased methylglyoxal concentrations have been observed in patients with diabetes and have been associated with progression of diabetic nephropathy [30]. Metformin, which has been used to lower elevated methylglyoxal concentrations in type 2 diabetic patients, was also able to prevent the development of sucrose-induced insulin resistance in rats [31,32]. Increased methylglyoxal has also been linked to impairments in insulin signaling in adipose tissue of fructose-fed rats [33]. Thus, several studies have suggested a link between the accumulation of methylglyoxal and glucose homeostasis.

Methylglyoxal can interact readily with certain arginine and lysine residues in proteins, leading to increased glycation of proteins and advanced glycation end products, such as N-epsilon-carboxyethyl-lysine and N-epsilon carboxymethyl-lysine [34,35]. In vascular smooth muscle cells, very high fructose concentrations (15 mM) increased methylglyoxal and peroxynitrite production, which was inhibited by reactive oxygen scavengers such as reduced glutathione or N-acetyl-l-cysteine [34]. In the present study, although fructose increased DCF fluorescence, amelioration via taurine did not result in a reduction of fructose-mediated stress signaling. Thus, the acute effects of fructose on methylglyoxal concentrations and fructose-mediated changes in stress and insulin signaling may operate independently of oxidative stress. It is important to emphasize that N-acetyl cysteine can act as both an anti-oxidant and a methylglyoxal scavenger [26,27].

Increased fructose consumption and therefore delivery appears to have multiple effects in vivo. Our data suggest that fructose may have rapid, direct effects on the liver that activate stress signaling pathways and reduce insulin signaling. We propose that these effects are largely mediated by local metabolism of fructose in the hepatocyte and perhaps generation of methylglyoxal. Future studies are needed to understand whether and how methylglyoxal mediates these fructose-induced changes. These hepatocyte-specific effects of fructose likely also contribute to lipid accumulation within the hepatocyte [3]. Recent studies have also demonstrated that fructose consumption in beverages or water can lead to accumulation of visceral fat and changes in intestinal barrier function [36,37]. Thus, overconsumption of sucrose and fructose can lead to adaptations in multiple organ systems. However, the quantitative contribution of sucrose and fructose consumption in foods and beverages to human metabolic diseases such as the metabolic syndrome and non-alcoholic fatty liver diseases remains unclear.

The cell system used in the present study, although offering several advantages, does not mimic the dynamic nature of in vivo dietary nutrient delivery. With this in mind, this cellular system was employed only after studies that demonstrated that diets enriched in sucrose or fructose, or fructose infusion in rats in vivo, increased stress signaling and reduced insulin signaling in the liver in vivo [3,6,38]. It is likely that the magnitude of hepatic stress induced by fructose will ultimately depend on the concentration presented to the liver, the duration of exposure to increased fructose delivery, as well as multiple biologic and perhaps genetic factors [8,39,40].

In the present study, we have examined the effects of physiologic concentrations of fructose on stress and insulin signaling in primary hepatocytes. Our results suggest that increase fructose delivery to hepatocytes modulates stress and insulin signaling. We hypothesize that fructose-mediated changes in methylglyoxal contribute to these acute effects.

## Abbreviations

JNK: c-jun NH_2_-terminal kinase; IRS-1: Insulin receptor substrate-1; IRS-2: Insulin receptor substrate-2; MKK: Mitogen activated protein kinase kinase; NAC: N-acetyl cysteine; RPMI: Roswell Park Memorial Institute media.

## Competing interests

The authors declare that they have no competing interests.

## Authors’ contributions

YW and DW helped design experiments, carried out experiments, analyzed data, edited manuscript. GM, AE carried out experiments. MJP conceived of the studies, carried out experiments, analyzed data and wrote the manuscript. All authors read and approved the final manuscript.
